# Crooked Timber: The life of Calvin Wells (1908–1978)

**DOI:** 10.1177/0967772013479734

**Published:** 2014-05

**Authors:** Tony Waldron

**Affiliations:** University College London, UK

**Keywords:** Calvin Wells, palaeopathology, cremations, pseudopathology, radiography

## Abstract

Calvin Wells was the leading palaeopathologist in the UK between the later 1950s and the early 1970s. He studied medicine at University College London but failed in anatomy and qualified in 1933 with the Conjoint Diploma (MRCS, LRCP). After qualification he began to study obstetrics and after war service in the RAMC he settled in Norfolk (UK), established a small general practice and took up palaeopathology. Although he was usually conservative in diagnosis he tended to over-interpret signs in the skeleton, often publishing descriptions that were more fiction than science. He held firm views on the way in which palaeopathology should be undertaken and in particular he resented the entry into the field of anthropologists without medical training. His major contributions to palaeopathology were related to the study of cremations and the introduction of the notion of pseudopathology, and his writings on these subjects have scarcely been improved upon since. He was extremely well read, warm and encouraging to those with archaeological or medical qualifications, but vituperative about those he disliked. His bone reports, which are a major proportion of his published output, generally were highly regarded but his writing is often marred by sexual innuendo and vulgarity which does his memory little credit.

## Introduction

During the late 1950s and throughout the 1960s and early 1970s Calvin Wells was the best known palaeopathologist working in Britain – indeed he would almost certainly have said, at least during the earlier years, that he was the *only* palaeopathologist working in the country by virtue of his medical qualification, essential in his view for this kind of work.

## Beginnings

**Figure 1.**
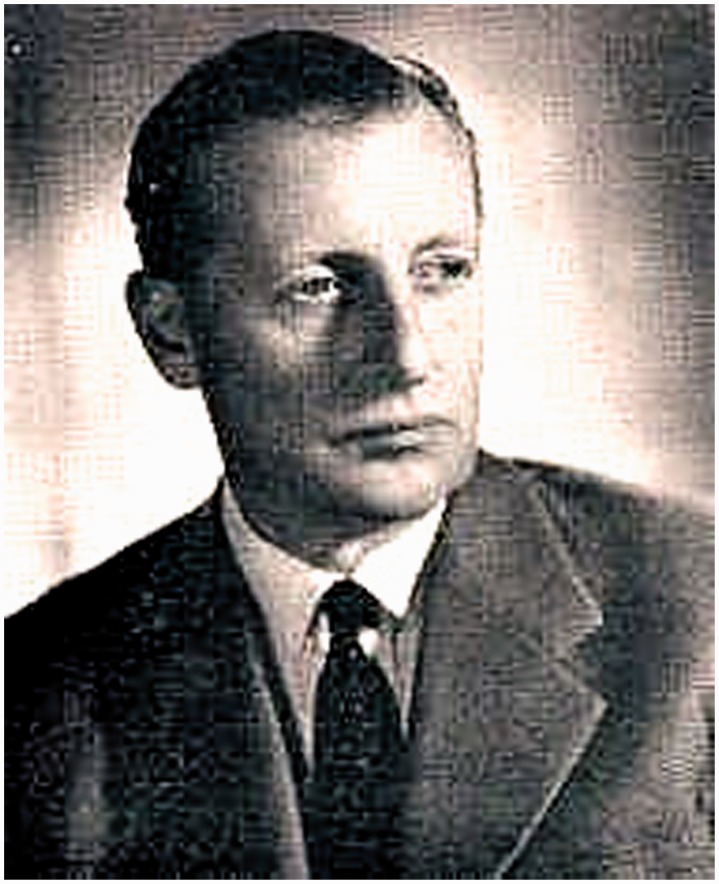
Calvin Wells (1908–1978). Images of Calvin Wells are scarce and further details of this image are not currently available.

Percival Calvin Bampfylde Wells (Figure 1) was born in Twickenham on 2 April 1908. From an early age he was known by his middle name – where Bampfylde came from is by no means clear since there seems to be no family connection on either side to the Bampfylde family. He was the first child of Arthur George and Violet Caroline Annie (always known to the family as Daisy, while Arthur came to be known – latterly at least – as Pops). Arthur Wells was both barrister and surgeon. He was a member of Gray’s Inn and had qualified in medicine from University College London (UCL) in 1906. He was Principal Assistant Medical Officer for London County Council (LCC), a JP and Honorary ENT Surgeon at the Downs Hospital for Children in Sutton, Surrey, an LCC Hospital. His medical career seems to have been both uneventful and unremarkable although he did publish one small book, *The discharging ear*, which he dedicated to his son.^1^ He survived into great old age, dying of coronary thrombosis in 1971 only seven years before Wells himself. Wells’ mother (née Heybourn), the daughter of a banker, was to die young from carcinoma of the ovary in 1929, when he was only 21 and still a medical student. Where Wells received his education is – like many facets of his life – somewhat unclear although apparently he attended many primary schools. His obituarists in both *The Times*^2^ and the *British Medical Journal*^3^ state that he was educated at Charterhouse, and so he was but only for two terms. During the Spring and Summer terms of 1922 when aged 14, he was a member of Daviessites House. There is no indication in the school archives, however, to explain why he was there for so short a time or his reason for leaving. What is certain, however, is that he followed his father to University College London (UCL) to study medicine when for at least part of the time he lived in Winchmore Hill in North London which provided relatively easy access to the college in Gower Street. Both obituarists mentioned suggest that during his time at UCL Wells came under the influence of Grafton Elliot Smith (1871–1937), Glyn Daniel (in *The Times)* actually stating that he studied anthropology under Elliot Smith, a notion that Wells put about himself and he was presumably Daniel’s source of information. Elliot Smith was certainly Professor of Anatomy at UCL during the time Wells was a student, having been appointed in 1919 and remaining there until he was forced to retire due to ill health in 1937 after Wells had qualified. Wells was never formally a student of anthropology at UCL although Elliot Smith may have taught him anatomy. Elliot Smith’s influence on his anatomical knowledge cannot have been great, however, since Wells failed the anatomy examination which meant that he could not proceed to the London medical degree (as his father had done) but had to qualify – in 1933 – with the Conjoint Diploma.

After qualification Wells took up the practice of obstetrics and held posts at Wimbledon Hospital and at Queen Charlotte’s Hospital then in the Marylebone Road and for a time he was Medical Superintendent at Barking Maternity Hospital where he was sometimes known as Dr Bampfylde-Wells, a form of address that occasionally was used at least until the late 1950s. He also held posts as Anaesthetist in the Department of Obstetrics at University College Hospital (UCH) where, in addition, he served as an Obstetric Assistant. The BMJ Obituary also mentioned that before the Second World War he had rooms in Wimpole Street and, if so, income from private practice might explain how in 1938 he came to be living in Devereux Court, a prestigious apartment block in the Strand from where he married Ida Clara Warrington in St Clement Danes Church in the Strand. Ida Warrington was then 31 years old, the daughter of an engineer living in Wimbledon in south London. This first marriage has either been ignored by or escaped the notice of Wells’ biographers, the usual story being that he had enjoyed 42 years as ‘the dearest companion, lover, friend, and husband of “Freddie”’^4^ – Winifred Petersen, a nurse whom he met at UCH some two years before his marriage to Ida; she was then a nurse at the hospital and he a houseman. The circumstances surrounding the marriage to Ida are obscure; there is no evidence that there were children from the marriage and there was no divorce since when she died in 1977 of Parkinson’s disease Ida was described on her death certificate as the ‘wife of Calvin Bampfylde Wells, doctor of medicine’. In fact, although Wells and Freddie adopted two children, a boy and a girl, they did not marry until 13 January 1978, most likely prompted by the news of Ida’s death and the knowledge of Wells’ own fatal illness.

During the Second World War, Wells served in the Royal Army Medical Corps, leaving the service with the rank of Captain. Throughout the war he was stationed in England, serving as Medical Officer variously in Ramsey, Norwich, Luton and, finally, in Colchester. It was during the war (in 1944) that Freddie, then living in Luton (in Bedfordshire) and working as a nurse in the Children’s Hospital, bought the Old Hall at Mulbarton – which Wells had come across during the time he was stationed in Norfolk – for the bargain price of £1600 (about £42,000 in today’s money). She signed her name on the conveyance as Winifred Petersen Wells indicating that the marriage to Ida had not survived long and that, although not married, Freddie had taken Wells’ name (by deed poll as it transpired). The Old Hall (or Manor House as it was sometimes referred to) was initially derelict and the Wells’ devoted much time and money to restoring it. They lived there until 1974 when, having given the Old Hall to their son, they moved into White Horse Cottage in Hapton (but still in Norfolk), a house they had converted some time before from the old White Horse pub.

## Post-war palaeopathology

Wells’ activities in the immediate post-war period are somewhat vague although he and Freddie settled in Mulbarton and he also established a single-handed general practice in Norwich, at 75 Thorpe Road. He deliberately kept his practice list small, hand picking his patients so they would be unlikely to want calls out of hours or inconvenience him in as few ways as possible so that he could devote most of his time to palaeopathology. His first post-war entry in *The Medical Directory* is in 1950 by which time he had become a Fellow of the Royal Anthropological Institute and where he gives reference to three papers (Chronology of the Egyptian 10th Dynasty; Anthropology and the teaching professions and Some aspects of the history of midwifery). All efforts to trace these publications have failed so presumably they did not appear in very prestigious journals. The 1951 *Medical Directory* entry shows Wells as now having a PhD although the title of his thesis is not given, nor is the university from which he obtained the degree; it is difficult to believe that it would have been from UCL since he had not obtained a first degree there, nor was he ever registered there as a post-graduate student. The thesis was not among his books when he died nor does it appear in the bibliography that Freddie compiled after his death, a serious omission on the face of it given the ferocity with which she guarded his reputation. A search for the thesis in the United Kingdom and in France, where Wells spent much time and where he had a house in later years, has failed to bring it, or the awarding body, to light and this is yet another of the curious features of his life. Since Wells was a considerable self-publicist it is strange that he was apparently so reticent about the thesis and it raises doubts as to whether there was a thesis at all.

Quite when Wells began to study human remains and what prompted him to do so again is unknown but his first publications – or, at least the first that are accessible – appeared in 1955 and he kept up a steady stream thereafter. There are two extensive bibliographies of his work, one containing 155 entries^5^ and the other 123 entries^6^ with, of course, considerable overlap between them but the former containing several early papers, for example articles in the *Nursing Mirror* and *The Listener*, that do not appear in the latter. In the early years Wells was virtually the only author from the UK publishing on palaeopathology to be cited in *Index Medicus* and there is no doubt that he carried the banner forward, albeit a somewhat grubby banner for several years, at least up until what Lawrence Angel (1915–1986) in the 1960s called the beginnings of modern palaeopathology.^7^

Wells was not at all enamoured with one of the most significant changes that took place in palaeopathology after the war – the preponderance of those without a medical degree who were now entering the field. From the earliest times, beginning in the late eighteenth century, almost all those who reported pathological change in human or fossil remains were medically qualified but after the war the great majority was without such a qualification, having mostly come to palaeopathology from a previous education or career in archaeology or anthropology. In a letter to Sonia Chadwick Hawkes (1933–1999) in 1973 Wells gave vent to his feelings:

My experience of anthropologists in general is that they tend to be too ‘cut and dried’ in their approach to anatomy. They suffer from not having spent years handling all the nuances, aberrations and mosaics of clinical material.^8^

One well-known anthropologist particularly irked him and in a series of letters to Sonia Hawkes and others he made several comments about him which were shockingly vituperative and frankly libellous, repeatedly referring to him as ‘the Rat’. By contrast he could be warm, helpful and encouraging to others who *were* medically qualified and he was particularly distressed when one of his favourite protégées, Merton Satinoff (1938–1972), died at the early age of 34.

After Wells’ death Freddie wrote to one of her correspondents that she saw his ‘diagnostic genius’ in operation many times. One adulatory biographer called him ‘the Sherlock Holmes of palaeopathology’^10^ but actually he was often somewhat conservative when making a diagnosis, stressing several times that the best diagnoses in palaeopathology are tentative ones. On the other hand his *interpretations* of what he saw were often fanciful in the extreme and, if he did not actually invent it, he did much to advance the cause of what might be best called the ‘interpretive’ school of palaeopathology. Certainly he used his imagination to the full and early publications introduce weavers, tailors^11^ and the village idiot^12^ to his readers, occupations all deduced from signs on their skeletons. He had expressed very strong views on the way in which the results of investigations should be presented and these were stated in his usual forthright manner in the introduction to his report on the bones from North Elmham in Norfolk:

The evidence [from bones] can be treated broadly speaking in two ways: either the most rigid restraint may be imposed on speculations based upon it or the imagination may be given freer rein to pursue more airy but less certain conjectures … This report does not hesitate to follow the second course.^13^

And follow it he did throughout his entire career. A later passage in the North Elmham report talks about the pathology in the skeletons that ‘goes far to disclosing their lives and habits to us … [B]y careful attention to the ills and accidents which plagued them, these people come to life in a way which no computerizing of their cranial contours could achieve’.^14^ Bringing past folk to life by looking at their bones was the touchstone of all his work, believing that the recitation of mere facts without elaborating their meaning to the living made for a dull read, and perhaps he was right for his reports were never that. Here, from an unpublished report, is part of his interpretation of his findings in a female skeleton from Piercebridge, County Durham:^15^

We can visualise a young woman, petite, well formed, perhaps overly curvaceous at the hips, but with neat hands and feet … In her youth she had been lithe and active but by the time we meet her she had withered perceptively and was no longer supple. Her back was slightly bowed, which flattened her chest, and her shrivelled breasts sag low. Her neck creaked as she turned her head with difficulty … [H]er hands were stiff and through her thin, blue-veined skin the shrunken interosseous muscles revealed her metacarpal bones, like the bars of a linnet’s cage.

There cannot be a single conjecture in this passage that can be validated, right down to the linnet’s cage (why a linnet?). It purports to put flesh on this woman’s bones but although it may be superficially appealing and, like most of Wells’ writings in the genre presented as fact, it is nothing more than the figment of his often overheated imagination; a nice piece of romantic fiction.

But there is a much less attractive theme that also runs through Wells’ work, a constant preoccupation with the sexual behaviour of those he wished to bring to life. Thus, when putting forward some ideas for a book on the theme of the doctor–patient relationship through the ages, he writes to the publisher that ‘For sales purposes keep it as (“respectably”) pornographic as possible – emphasis on details of early gynaecology, contraception, etc. Illustrate with early obstetrics and primary things like amputations, castrations (penal and ecstatic, etc)’.^16^ On an undated postcard to Sonia Hawkes he asks of a female skeleton from Kingsworthy in Hampshire ‘Why didn’t you tell me that Inh 78 had been raped?’ and there follows a rather unpleasant exchange of views on the amusing nature of the act. The flimsy evidence on which the diagnosis of rape was based was later jointly published in *Antiquity*.^17^ Wells continues his description of the woman from Piercebridge by saying:

At times she hobbled slightly when she walked, less from her aching back than from the screws in her left foot – the legacy of a sprain when, a giggling girl, she had jumped from the granary ladder or romped with Agricola’s randy son at haysel or harvest … [H]er health had always been good until these last years  …  The most she could remember … was shrieking three children into the world. But thanks to Lucina and a broad pelvis, even her first labour was accomplished in less than sunset to sunset, while her wide pubic arch had saved her perineum from splitting more deeply than the breadth of a lecherous eye.

The final paragraph of the North Elmham report referred to above is remarkable both for its invention and for its vulgarity, both of which are present in roughly equal measure and the language is so explicit that it is a surprise it was published without alteration.

Not that anyone would lightly touch the prose of which he was so proud. On one occasion he wrote a three and a half page rant to the Director of the Suffolk Archaeology Unit because someone had ‘deliberately altered one word’ of his report on two burials from a tumulus at Barrow Bottom, Risby, in Suffolk: ‘Need I tell you that the result has been to change a clear, grammatically correct sentence into an ugly solecism? … I am almost apoplectic with rage. I have never disguised … I would infinitely prefer to be judged an incompetent pathologist than write bad English’.^18^ He demanded that there should be an apology in the next volume of the *East Anglian Archaeology Reports* in which the offending report had appeared, that the mistake be amended in all stock copies, and he threatened never to work for the unit again. And the outrage that been committed? To alter a single word from the original which noted that ‘There is no caries’ to, ‘There are no caries’. The normal response to this egregious error would have been to sigh gently and wish that everyone knew that caries was a singular noun but Wells had to show off by lecturing his correspondent: ‘As you know [meaning, of course, as *I* know and *you* do not] this Latin word meaning decay occurs only in the nominative, accusative and ablative singular’. Needless to say, although he received a grovelling apology, there was no correction and the error persists, buried deep on the second page of a five page report.^19^

The recipients of Wells’ bone reports that form a large part of his published output were often extremely satisfied with what they got. ‘Very many thanks for your, as usual, “spiffing” report’, enthuses Miss AS Mottram, Curator of the King’s Lynn Museum and Art Gallery,^20^ ‘I … am delighted that you get so much information out of [the bones]’.^21^ Elizabeth Owles, an archaeological assistant at the Ipswich Museums and Art Galleries, likewise is enthusiastic: ‘Thank you for the Boxford report, it’s marvellous what you make of these beastly fragments’^22^ and a week later, presumably having read or reread the report, she writes ‘Yours are the only specialist reports I know which are interesting and amusing; most of them are utterly unintelligible and dull’.^23^

Wells also wrote extensively for non-medical journals under his own name and a variety of pseudonyms but only a handful of those written under his own name has been tracked down. His writing was undertaken to boost his income from palaeopathology after giving up his general practice sometime in the late 1960s. Some archaeologists were surprised that he made a charge for looking at human remains. For example Tony Rook wrote to Wells in 1967 to ask whether he would look at some Romano-British cremation, ‘What fee?’ Wells wrote on the letter and then presumably wrote to ask. ‘No-one has asked for money before’ Rook replied to which Wells replied that he was the only palaeopathologist who looked at cremations and a fee of £10 was required, whether for each cremation or for all of them is unclear although it was probably the former.^24^ Wells’ request for fees sometimes provoked comments from other sources too. John Musty (1923–2000), writing to Sonia Hawkes about difficulties with the bones from Worthy Park in Hampshire, noted that Wells had asked for £100 a skeleton to produce a report for another site, a figure so alarming that the Ancient Monuments’ Laboratory had been forced to take up matters with the British Museum who undertook to work for free.^25^ We should not be too hard on Wells though for he had a family to support, a ruin of a house to keep up and his income from his practice was (deliberately) limited, hence his need to be paid for his bone work and for his forages into other sorts of writing. It should also be remembered that others who might perhaps compete with him for work were almost all in paid employment, in university or hospital departments, attracting regular and usually not immodest salaries. Wells did have one useful source of income however, for he received money from the estate of his mother’s brother, Percy Heybourn, who left the bulk of his fortune in trust. The money had been mismanaged by the trustees so that, although eventually he received £30,000 pounds, this was substantially less than the original sum.

## Wells’ contributions

The somewhat critical account of Wells’ activities thus far should be tempered by recognising his positive achievements. His well-known book, *Bones, Bodies and Disease* published in 1964, was an extremely lively introduction to palaeopathology, emphasising that evidence for disease in the past should be looked for in places other than simply human remains.^26^ There is no doubt the book stimulated interest in palaeopathology and generally was well received by its reviewers although one did not like ‘the few not too happy attempts at fine writing’ but who could not then, himself, resist showing off by quoting some lines from Horace to end his piece.^27^ The book sold sufficiently well to go into a second printing the year after it was published and it brought Wells some much appreciated royalties.

On the more specialised front, Wells’ most significant contributions were to promote the study of cremations, to introduce the concept of pseudopathology and to encourage the use of radiography in palaeopathology.

Despite the fact that they were the most frequent means of disposing of the dead at some periods and in some cultures, cremations attracted scarcely any attention from those studying human remains, Nils-Gustaf Gejvall (1911–1981) being almost the only author to do so in the immediate post-war period. His earliest publications were in Swedish which meant that they were not easily accessible to English speaking anthropologists. Wells, who was fluent in French and who was reputed to be able to read most modern European languages, may have been one of the few who could read Gejvall’s papers; he certainly cites three of them in his own paper on cremation.^28^ Wells built on Gejvall’s work and showed it was possible to age and sex at least some cremated remains with a reasonable degree of certainty and that pathological change could sometimes be observed, and he was able to suggest how the body might have been placed on the pyre (beneath it or above it) by studying the pattern of calcination of the various skeletal elements. He supplemented his observations on the archaeological remnants with visits to modern crematoria where not only was he able to watch proceedings as they occurred but he could also persuade the operators to experiment with the position of the body within the furnace to test some of his hypotheses. The importance of his work on cremations is such that very little has been added to it in the half century or so since it first appeared, and nowadays routinely cremations are examined and reported on using techniques that have changed little since his time.

Pseudopathology was a concept that Wells introduced in the first chapter of *Diseases in antiquity* edited by Don Brothwell and Andrew Sandison (1923–1982), the publication of this book being one of the three events that Angel considered as having ushered in the modern era of palaeopathology. In this chapter^29^ Wells discussed the ‘lesions’ that might be produced in bone by agents as diverse as bacteria, fungi, soil erosion, roots, boring beetles, gnawing animals and, by no means the least of them all, the act of excavation itself. He also counselled against mistaking normal variation for pathology, a skill that could be gained only from the examination of very many skeletons. Students and novices in the field are still apt to be led astray by pseudopathological change and Wells’ chapter should be required reading for all those who begin the study of human remains. As with cremations, little of substance has been added to Wells’ original, perceptive comments.

## Radiology

Wells’ writings on radiology were of less importance than on the previous two topics. There were many pioneers of radiology in palaeopathology and the technique had actually been applied to the study of human remains soon after Roentgens’ discovery of X-rays in 1895. Elliot Smith, for example, records taking the mummy of Thutmose IV in a taxi to a private nursing home in Cairo 1904 – with his feet sticking out of the window, one supposes – to have his picture taken. In the 1920s Roy Moodie (1880 –1934) published one of the first comprehensive radiographic studies of Egyptian and Peruvian mummies in the Field Museum in Chicago.^30^ Although Wells was by no means leading the charge, he found that radiography had been sadly neglected by palaeopathologists and he set out to correct this in a chapter in another book edited by Brothwell (this time with Eric Higgs (1908–1976) as co-editor), *Science in archaeology*, published in 1963.^31^ Wells mentioned some of the more obvious uses of radiography, namely to examine structures within the bone which could not otherwise be visualised, to make or confirm diagnoses and to examine mummies without the necessity of unwrapping them and thereby inevitably damaging them. Wells took his own X-rays using a portable apparatus given to him by an indulgent manufacturer. The machine was kept in one of his outbuildings with scant regard for matters of health and safety, but then Wells generally had little time for what he viewed as pettifogging bureaucracy

## Wells’ death and afterwards

Apart from an attack of Menière’s disease that may have resulted from a water-skiing accident and which prevented him from accepting an invitation to deliver the Grogan Lecture at the Toronto Academy of Medicine in 1976, Wells had no serious illnesses until 1977 when he was discovered to have very aggressive prostatic carcinoma. At the time of diagnosis he was planning an international meeting on Disease in Ancient Man to be held at the Royal Society of Medicine in London in 1979, a meeting he was never to attend. He had an operation in October 1977 followed by two further surgical procedures and radiotherapy but despite treatment he quickly developed skeletal secondaries. According to Freddie ‘He was… very brave … and faced his approaching end with great equanimity and fortitude … It was a terrible time for him’,^32^ so terrible that he committed suicide by taking sodium amytal on 31 July 1978, he and Freddie having finally been able to marry a few months previously. An inquest was held in Diss the following week but the records of the proceedings subsequently were lost.

After Wells’ death Freddie began a strenuous, not to say aggressive, campaign to see his unpublished reports into print and to collect a complete collection of his published works. In the latter respect she became obsessive, bombarding Wells’ ex-colleagues for reprints and berating those who had neglected to order any copies of articles of which Wells was a joint author. Her badgering became so persistent that she was in danger of giving offence. For Sonia Hawkes she seems to have succeeded:

This is really becoming the outside of enough! With every letter and every offprint you send me you rage and abuse me. If you treat every one of Calvin’s archaeological colleagues in this fashion can you wonder that some of them choose to ignore you? … {T]hese personal recriminations go beyond reasonable bounds. You are doing Calvin’s cause a disservice by these excesses.^33^

But, as she wrote to John Musty, Freddy felt that she *had* to bother

otherwise people … publish his work … and when I do eventually find out I have left with NO COPY and … often find errors which could have been avoided had I been consulted and allowed to proof read.^34^

Freddie also undertook the work of arranging Wells’ voluminous correspondence, his collection of some 6500 reference cards and his 1500 slides. His medical books, and those of his father, were offered to the Sir Thomas Browne Library at the Norfolk and Norwich Hospital but it seems that the offer was declined since in 1984 they were donated to the Department of Archaeological Sciences at the University of Bradford together with his journals, papers, reference cards and slides, the books and papers later being moved to the University’s JB Priestley Library. The University established the Calvin Wells Laboratory following the receipt of Wells’ effects only for it to be renamed sometime later as the Biological Anthropology Research Centre. Freddie died herself in 1988 at the age of 88 and this brought Wells’ extended career in palaeopathology to an end.

## Conclusion

There is no doubt that Wells was a significant figure in palaeopathology. He promoted the subject through his writing and through lectures to both lay and medical audiences, and he tried to establish courses in palaeopathology in Oxford and London (at Guy’s Hospital) in the 1960s but without success. He was extraordinarily well read in several languages and he had correspondence with many workers throughout the world. He welcomed people to his house so long as they were archaeologically or medically qualified. Generally he was considered amusing and convivial, and something of a bon viveur. He had widespread interests including a belief in extrasensory perception and telepathy that he shared with Freddie. But he was also vitriolic about those he disliked; he abandoned his first wife and displayed a lewdness in some of his writings which does his memory little credit. His over-interpretation of findings in the skeleton seems now to be somewhat passé but it pleased many of his readers and his influence on palaeopathology and palaeopathologists was, and in some quarters remains, substantial. He truly was many things to many people and his life perhaps exemplifies Kant’s observation that from the crooked timber of humanity nothing straight was ever made.
